# Single-Cell Transcriptomic Profiling Reveals That Macrophage-Induced Angiogenesis Contributes to Immunotherapy Resistance in Hepatocellular Carcinoma

**DOI:** 10.3390/biology15010095

**Published:** 2026-01-02

**Authors:** Xinyu Pan, Baolin Liao, Zhijie Hu, Yuanyan Xiong

**Affiliations:** 1 Key Laboratory of Gene Engineering of the Ministry of Education, Department of Biochemistry, School of Life Sciences, Sun Yat-sen University, Guangzhou 510275, China; merakp0029@gmail.com; 2 Department of Hepatology, Guangzhou Eighth People’s Hospital, Guangzhou Medical University, Guangzhou 510440, China; polinlbl@163.com; 3 Guangzhou Key Laboratory of Clinical Pathogen Research for Infectious Diseases, Guangzhou 510440, China

**Keywords:** hepatocellular carcinoma, immune checkpoint blockade, M0 macrophages, tumor angiogenesis, transcriptional regulation

## Abstract

Hepatocellular carcinoma (HCC) ranks among the top causes of cancer-related mortality worldwide, and its complex tumor microenvironment (TME) contributes to poor responses to immunotherapy. Although PD-1/PD-L1 blockade has emerged as an effective treatment strategy, therapeutic resistance frequently limits its clinical benefit. Here, we uncover a distinct macrophage population associated with anti-PD-1 resistance in HCC. Single-cell transcriptomic profiling reveals an *NFKBIZ*^+^ M0 macrophage subset predominantly present in non-responders. Notably, these macrophages exhibit a hypoxia-induced phenotype characterized by the secretion of *VEGFA* and *HBEGF*, which cooperatively enhance tumor angiogenesis, alongside an elevated expression of the inflammatory chemokines *CXCL2*, *CXCL3*, and *CXCL8* that consolidate an immunosuppressive, pro-tumorigenic microenvironment. Transcriptional regulatory network analysis further identified *FOSB*–*VEGFA* and *FOS*–*HBEGF* axes as key drivers of this pathogenic macrophage phenotype. Our findings define a distinct *NFKBIZ*^+^ macrophage population that mechanistically links hypoxia, angiogenesis, and immune evasion to PD-1 blockade resistance. This work provides new insights into the cellular and molecular basis of immunotherapy failure in HCC and highlights potential targets for overcoming treatment resistance.

## Simple Summary

Hepatocellular carcinoma (HCC) is one of the leading causes of cancer-related deaths worldwide, and many patients do not respond well to immunotherapy. In this study, we identified a unique group of macrophages—called *NFKBIZ*^+^ M0 macrophages—that are enriched in patients non-responsive to anti-PD-1 treatment. These macrophages are activated by hypoxia and release factors such as *VEGFA* and *HBEGF* that promote new blood vessel formation and tumor growth. They also produce inflammatory molecules that suppress the immune system, helping the tumor evade immune attack. Further analysis revealed that specific signaling pathways (*FOSB*–*VEGFA* and *FOS*–*HBEGF*) drive this harmful macrophage behavior. Our findings uncover a new mechanism linking hypoxia, angiogenesis, and immune evasion to treatment resistance in HCC and suggest potential therapeutic targets to improve immunotherapy outcomes.

## 1. Introduction

Hepatocellular carcinoma (HCC) is the sixth most common malignancy and the third leading cause of cancer-related death worldwide [1]. Its pathogenesis is intricately linked to chronic liver disorders, such as viral hepatitis, alcoholic liver disease, and metabolic-associated fatty liver disease [1,2,3]. Substantial progress has been achieved in HCC diagnosis and treatment, including surgical resection, liver transplantation, local ablation, systemic therapies (such as targeted agents and immune checkpoint inhibitors), and emerging precision medicine approaches [1,4]. The high heterogeneity and acquired drug resistance of HCC remain major barriers to improving clinical outcomes [4,5].

Tumor immune escape is a hallmark of cancer progression, with immune checkpoint molecules playing a pivotal role in dampening anti-tumor immunity. Among them, the interaction between programmed cell death protein 1 (PD-1) and its ligands (PD-L1/PD-L2) is well characterized: PD-1 binding to PD-L1 suppresses T-cell cytotoxicity, fostering immune inhibition and enabling tumor immune escape [6,7,8,9]. This pathway has thus become a cornerstone of cancer immunotherapy. Monoclonal antibodies targeting PD-1, such as Nivolumab and Ipilimumab, have demonstrated meaningful clinical efficacy in HCC [10,11]. However, their utility is limited by two critical challenges, immune-related adverse events [12,13] and a modest response rate in many patients [14,15,16], highlighting an urgent need to unravel the underlying mechanisms of immunotherapy resistance.

Tumor angiogenesis is another key driver of malignant tumor growth, invasion, and metastasis, involving complex crosstalk between multiple cell types, signaling pathways, and tumor microenvironment (TME) remodeling [17]. The hypoxia-induced secretion of pro-angiogenic factors (e.g., *VEGF*, *PDGF*) activates endothelial cells, triggering neovascularization through processes including endothelial cell migration, lumen formation, and vascular maturation [17,18]. Specifically, *VEGF-A* promotes angiogenesis via *VEGFR2*-mediated PI3K-AKT and ERK signaling, pathways targeted by clinically approved anti-angiogenic therapies [19].

The TME is a dominant determinant of immune checkpoint blockade (ICB) efficacy, with tumor-associated macrophages (TAMs) and their precursor cells being the most abundant myeloid population in many solid tumors [20]. TAMs exhibit functional plasticity, primarily polarizing into two phenotypes: M1 macrophages, which secrete pro-inflammatory cytokines and support anti-tumor immunity [21,22], and M2 macrophages, which release anti-inflammatory mediators to suppress CD8^+^ T-cell function, recruit regulatory T cells, and promote tumor immune evasion [21,22,23]. Beyond M1/M2 polarization, unpolarized M0 macrophages also exist in the TME and are closely associated with tumor progression and poor prognosis [24,25,26]. Pan-cancer analyses have further identified macrophage migration inhibitory factor (*MIF*), which serves as a marker of M0 macrophages, as a strong correlate of tumor-related immunosuppression [27]. Although the importance of M0 macrophages in the tumor microenvironment has been recognized, their precise roles and molecular mechanisms in mediating ICB resistance in HCC remain poorly defined.

To address this knowledge gap, we investigated the impact of M0 macrophage infiltration on anti-PD-1 therapy resistance in HCC. We integrated single-cell RNA sequencing (scRNA-seq) data from 328 samples of 63 HCC patients receiving anti-PD-1 treatment. Our analyses identified an *NFKBIZ*^+^ M0 macrophage population specifically enriched in non-responders to ICB. Mechanistically, this subset responds to hypoxia by expressing *VEGFA* to directly promote tumor angiogenesis while highly expressing the chemokines *CXCL2*, *CXCL3*, and *CXCL8* to establish a chronic inflammatory microenvironment that indirectly fosters tumor progression and vascularization. Furthermore, we revealed that *FOSB*–*VEGFA* and *FOS*–*HBEGF* regulatory axes cooperate within *NFKBIZ*^+^ macrophages to drive angiogenic signaling and enhance immunotherapy resistance. Our study defines a distinct macrophage population and elucidates its dual pro-angiogenic and pro-inflammatory roles in shaping ICB resistance in HCC, providing novel insights into the cellular basis of treatment failure and identifying potential therapeutic targets to improve immunotherapy outcomes.

## 2. Materials and Methods

### 2.1. Data Collection

Single-cell transcriptomic data were retrieved from the European Genome-phenome Archive (EGA; accession number: EGAS00001007547) and the National Center for Biotechnology Information (NCBI) Gene Expression Omnibus (GEO; accession number: GSE206325) [28,29]. Samples that had not received anti-PD-1 treatment were excluded from the analysis. Metadata fields were harmonized to ensure consistent annotation across all datasets. RNA-seq count data and clinical information for LIHC were obtained from the GDC Hub of UCSC Xena (https://xenabrowser.net/datapages/), accessed on 2 December 2024, and we reversed the log_2_(count + 1) transformation to recover the raw counts.

### 2.2. Normalization, Feature Selection, Scaling, Dataset Integration, and Batch Effect Correction

All datasets were integrated and processed using Seurat (v5.0.3) [30]. First, datasets were normalized using the NormalizeData function, and the top 3000 highly variable genes (HVGs) were identified using the FindVariableFeatures function. Data were scaled with the ScaleData function, followed by dimensionality reduction performed using the RunPCA function. Subsequently, all datasets were merged into a single Seurat object, with redundant assays and metadata discarded. The integrated dataset was subjected to reprocessing, including normalization, variable gene selection, scaling, and PCA. Batch effects were corrected using Harmony (v1.2.0) [31], with sample ID designated as the batch variable.

### 2.3. Cell Type Annotation and Marker Gene Identification

Cell type annotation was performed using SingleR (v2.2.0) [32], with clusters annotated to major cell types based on the tool’s output. Cell type-specific marker genes were identified using COSG (v0.9.0) [33], and the top 50 marker genes per cell type were selected based on the RNA assay and log-normalized expression values. In addition, canonical marker genes for each lineage were curated based on prior biological knowledge. Both the automated annotations and identified marker gene were further refined by manual inspection. Cell type assignments were corrected as necessary according to marker gene expression profiles, ensuring maximal annotation accuracy.

### 2.4. Gene Set Scoring

Gene set scoring was performed using a predefined angiogenesis-related gene set (including *VEGFA*, *HBEGF*, *FST*, *PDGFB*, *TGFB1*, *CXCL1*, *CXCL2*, *CXCL3*, and *CXCL8*) [34,35,36,37,38]. The AddModuleScore function in Seurat [30] was applied to calculate per-cell activity scores for this gene set.

### 2.5. Consensus Non-Negative Matrix Factorization (cNMF) Analysis

To decipher macrophage functional modules, consensus non-negative matrix factorization was performed using cNMF (v1.6.0) [39]. Filtered count matrices were exported and analyzed with default parameters, with k values tested ranging from 2 to 10. The optimal number of components (k = 7) was determined based on the k-selection plot. cNMF loadings were imported into Seurat [30], and cell clusters were annotated by dominant usage patterns. Marker genes for each functional module were identified using COSG [33].

### 2.6. Differential Gene Expression (DEG) and GO Enrichment

DEGs between groups (e.g., treatment efficacy, cell type, or state) were identified using Seurat’s FindMarkers function, with the Wilcoxon rank-sum test and false discovery rate (FDR) correction applied. Genes with adjusted a *p*-value < 0.01 and |avg_log_2_FC| > 0.2 were considered significant. GO Biological Process (BP) functional enrichment analysis was conducted using clusterProfiler (v4.2.2) [40].

### 2.7. Weighted Gene Co-Expression Network Analysis (hdWGCNA)

To explore gene co-expression modules, hdWGCNA (v0.3.03) [41] was applied to selected cell subsets (e.g., macrophage subclusters). Genes expressed in >1% of cells were used to construct metacells, followed by data normalization and the construction of signed co-expression networks with a soft-thresholding power of 6 and subsequent Topological Overlap Matrix (TOM) calculation. GO enrichment analysis for each module was performed using enrichR (v3.4).

### 2.8. Trajectory and Pseudotime Analysis

Monocle2 (v2.28.0) [42,43,44] was employed to reconstruct cell state transitions and pseudotime ordering for selected macrophage populations. The top 200 DEGs (ranked by adjusted *p*-value and logFC) between efficacy groups were used as ordering genes. Dimensionality reduction was performed via the DDRTree algorithm, and cells were assigned to trajectory branches and pseudotime ordering.

### 2.9. Gene Regulatory Network Inference (pySCENIC)

Gene regulatory networks (GRNs) were reconstructed using pySCENIC (v0.12.1) [45]. Cell-level expression matrices were exported, and GRNs were inferred using the GRNBoost2 algorithm. This was followed by motif enrichment analysis and regulon activity (AUCell) score.

### 2.10. GSVA-Based Immune Signature Scoring

Single-sample Gene Set Variation Analysis (ssGSEA) was performed using GSVA (v1.48.3) [46] with parameters set as follows: method = “ssgsea”, kcdf = “Gaussian” and abs.ranking = TRUE. This analysis was used to calculate enrichment scores of predefined immune gene sets [47] for each patient. The resulting score matrix was transposed and merged with clinical metadata using standardized patient identifiers.

### 2.11. Survival Analysis

For each cancer type, patients were stratified into high- and low-expression groups based on the angiogenesis-related M0 macrophage signature, with stratification thresholds set as the median ssGSEA score. Kaplan–Meier survival analysis was conducted using the survival (v3.5-7) and survminer (v0.5.0) packages. Log-rank *p*-values and corresponding risk tables were reported using the ggsurvplot function.

### 2.12. Immunohistochemistry Data Acquisition

Immunohistochemistry (IHC) images for VEGFA and CXCL8 were obtained from the Human Protein Atlas (HPA; https://www.proteinatlas.org). The protein expression of these two molecules in normal liver tissue, HCC, and cholangiocarcinoma was examined using publicly available IHC datasets generated by the HPA consortium. The antibodies used for IHC staining were CAB039240 (for VEGFA) and HPA057179 (for CXCL8). Representative IHC images from individual patients were selected for analysis, with patient IDs 3402, 2429, 2766, 3324, 2279, 3334, 1720, and 3196. All images were generated using validated antibodies and standardized staining protocols as described by the HPA.

### 2.13. Visualization

Visualization was performed using the following software packages: Seurat (v5.0.3), Monocle 2 (v2.28.0), ggplot2 (v3.4.4), cowplot (v1.1.1), ggpubr (v0.6.0), ggrepel (v0.9.4), pheatmap (v1.0.12), GOplot (v1.0.2) and survminer (v0.5.0) in R; cNMF (v1.6.0) was used for visualization in Python (v3.10).

### 2.14. Statistics and Reproducibility

Statistical significance was defined as a *p*-value < 0.05. All statistical analyses were performed in the R (v4.1.3) environment with RStudio (“Elsbeth Geranium” Release). For single-cell data, DEG analyses were performed using the Wilcoxon rank-sum test. Survival differences were evaluated using Kaplan–Meier analysis with the log-rank test. The code for analyses is provided in the Data and Code Availability section to facilitate the reproducibility of the results.

## 3. Results

### 3.1. Myeloid Cells Associated with Tumor Angiogenesis in HCC

**Figure 1:**
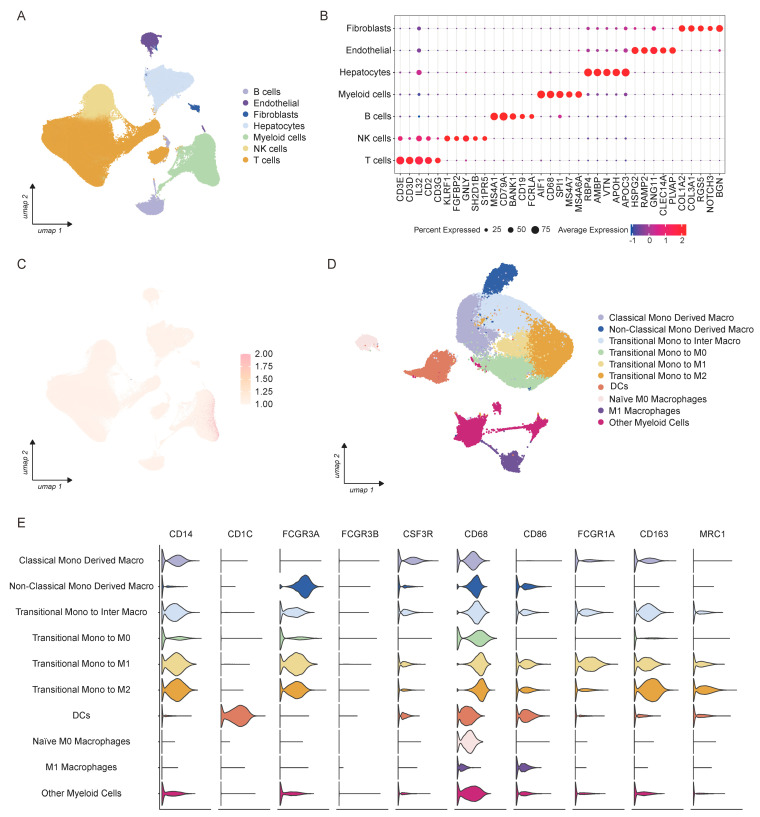
The single-cell transcriptomic landscape of HCC patients treated with anti-PD-1 therapy. (**A**) A UMAP visualization of sc-RNA-seq data showing major cell types identified across samples, including T cells, NK cells, B cells, myeloid cells, hepatocytes, endothelial cells, and fibroblasts. (**B**) A dot plot illustrating the average expression and percent of cells expressing gene signatures across annotated cell types. (**C**) A feature plot showing the score distributions of the angiogenesis-related gene set across cells. (**D**) A UMAP visualization of myeloid cells showing cell types, including Classical Mono Derived Macro, Non-Classical Mono Derived Macro, Transitional Mono to Inter Macro, Transitional Mono to M0, Transitional Mono to M1, Transitional Mono to M2, DCs, naïve M0 macrophages, and M1 macrophages. (**E**) Violin plots showing the expression distribution of representative marker genes (*CD14*, *CD1C*, *FCGR3A*, *FCGR3B*, *CSF3R*, *CD68*, *CD86*, *FCGR1A*, *CD163*, and *MRC1*) across distinct myeloid cell subsets.

HCC remains a major global health challenge, characterized by high intratumoral heterogeneity and an immunosuppressive TME that impedes therapeutic efficacy. To identify myeloid cell populations driving tumor angiogenesis in HCC with a specific focus on the context of PD-1 inhibitor treatment, we performed integrative scRNA-seq analysis on 328 samples from 63 HCC patients who received anti-PD-1 therapy. Patients were stratified into two groups based on treatment response: responders (pathological complete response, pCR) and non-responders (non-pCR). After rigorous quality control, 393,428 high-quality single cells were retained for subsequent clustering analysis. Unsupervised clustering revealed seven major cell populations in the HCC TME, T cells, natural killer (NK) cells, B cells, myeloid cells, hepatocytes, endothelial cells, and fibroblasts (Figure 1A,B), which aligns with the cellular composition reported in previous HCC scRNA-seq studies [48]. To pinpoint cell populations linked to angiogenesis, we used a predefined angiogenesis-related gene set (including *VEGFA*, *HBEGF*, *FST*, *PDGFB*, *TGFB1*, *CXCL1*, *CXCL2*, *CXCL3*, and *CXCL8*) Based on previously published studies, we selected an angiogenesis-related gene set (*VEGFA*, *HBEGF*, *FST*, *PDGFB*, *TGFB1*, *CXCL1*, *CXCL2*, *CXCL3*, and *CXCL8*) [34,35,36,37,38] to calculate scores for each cell. Notably, myeloid cells exhibited significantly higher angiogenesis scores than all other cell populations, with the highest enrichment concentrated within this lineage (Figure 1C). We further focused on the myeloid cell compartment, which comprised 45,688 cells. Following unsupervised subclustering and manual refinement based on canonical marker genes, we annotated nine distinct myeloid subsets (Figure 1D). The majority of these subsets co-expressed both monocyte and macrophage markers (Figure 1E and Figure S1), indicating a transitional state between monocyte recruitment and macrophage polarization. According to their unique expression profiles, we classified these subsets into six transitional populations: Classical Mono Derived Macro, Non-Classical Mono Derived Macro, Transitional Mono to Inter Macro, Transitional Mono to M0, Transitional Mono to M1, and Transitional Mono to M2. Additionally, small fractions of dendritic cells (DCs), naïve M0 macrophages, and classically activated M1 macrophages were identified in the myeloid compartment (Figure 1E).

### 3.2. Single-Cell Landscape Identifies Cell Subsets Linked to ICB Non-Response

**Figure 2:**
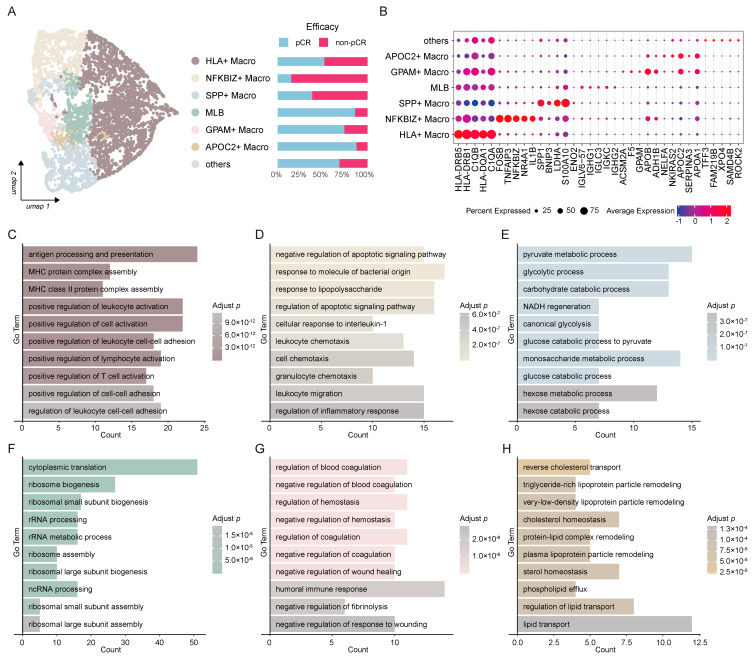
Characterization of Transitional Mono to M0 subset. (**A**) UMAP projection highlighting six cell types, including *HLA*^+^ Macro, *NFKBIZ*^+^ Macro, *SPP*^+^ Macro, MLB, *GPAM*^+^ Macro, and *APOC2*^+^ Macro, with corresponding barplot showing their proportion in pCR vs. non-pCR groups. (**B**) Dot plot illustrating average expression and percent of cells expressing gene signatures across annotated cell types. (**C**–**H**) GO biological process (BP) enrichment analysis of top 100 marker genes from (**C**) *HLA*^+^ Macro, (**D**) *NFKBIZ*^+^ Macro, (**E**) *SPP*^+^ Macro, (**F**) MLB, (**G**) *GPAM*^+^ Macro, and (**H**) *APOC2*^+^ Macro.

To explore the impact of early-stage macrophage states in the TME on ICB efficacy, we performed a subclustering analysis of Transitional Mono to M0 cells using consensus non-negative matrix factorization (cNMF). Seven components were selected for downstream analysis based on the inflection point in stability curves and error metrics (Figure 2A and Figure S2A,B). This cNMF-based subclustering identified six distinct subpopulations, designated as *HLA*^+^, *NFKBIZ*^+^, *SPP*^+^, macrophage-like B cells (MLBs), *GPAM*^+^, and *APOC2*^+^, according to their characteristic marker genes (Figure 2B). Consistent with previous findings [49], *SPP*^+^ macrophages were predominantly enriched in the non-pCR group (61.6%) (Figure 2A), supporting their potential pro-tumorigenic function. Notably, the *NFKBIZ*^+^ macrophage subset showed the strongest association with ICB resistance, with 84.9% of these cells enriched in non-pCR group (Figure 2A), highlighting its critical role in mediating ICB tolerance. In contrast, *HLA*^+^ macrophages were relatively evenly distributed, with 51.9% localized in the pCR group (Figure 2A). The remaining three subpopulations, namely MLB (86.0%), *GPAM*^+^ macrophages (74.2%), and *APOC2*^+^ macrophages (87.7%), were significantly enriched in the pCR group (Figure 2A), suggesting potential anti-tumor or ICB-sensitizing roles.

Functional enrichment analysis revealed distinct biological roles for each subset: specifically, *HLA*^+^ macrophages were mainly associated with immune antigen processing and presentation, as well as lymphocyte and leukocyte activation, reflecting canonical macrophage immune functions (Figure 2C); *NFKBIZ*^+^ macrophages were linked to chemotaxis, the negative regulation of apoptosis, and inflammation modulation (Figure 2D); *SPP*^+^ macrophages exhibited an enrichment of glucose metabolic pathways with a particular focus on glycolysis (Figure 2E), which is consistent with previous observations that TAMs in HCC promote tumor progression and suppress T-cell function through enhanced glycolysis [50]; the MLB population displayed high protein synthesis activity (Figure 2F), consistent with its B-cell-like phenotypic features [51]; *GPAM*^+^ macrophages highly expressed genes involved in the negative regulation of blood coagulation and humoral immune responses (Figure 2G); and *APOC2*^+^ macrophages expressed genes mainly participating in lipid and lipoprotein metabolism (Figure 2H).

### 3.3. HBEGF, VEGFA, and Members of the CXCL Family Play Essential Roles in Macrophage-Mediated Angiogenesis

**Figure 3:**
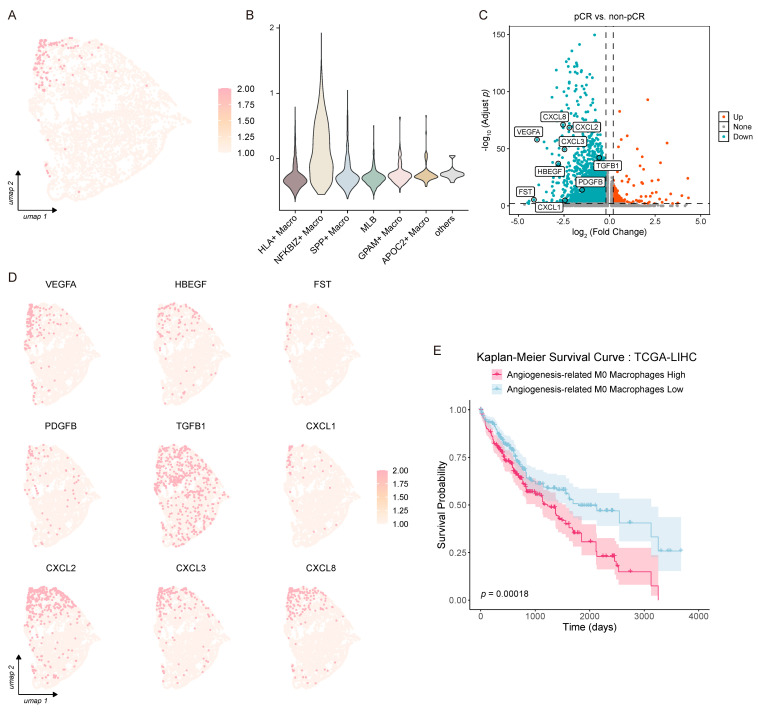
Pro-angiogenic M0 macrophages associated with ICB resistance in HCC. (**A**) A feature plot showing the score distributions of the angiogenesis-related gene set across Transitional Mono to M0. (**B**) A vlnplot showing the score distributions of the angiogenesis-related gene set across Transitional Mono to M0 subsets. (**C**) A volcano plot showing representative differentially expressed genes; genes from the angiogenesis-related gene set are labeled in the plot. (**D**) Feature plots showing the expression distribution of the angiogenesis-related gene set (*VEGFA*, *HBEGF*, *FST*, *PDGFB*, *TGFB1*, *CXCL1*, *CXCL2*, *CXCL3*, and *CXCL8*) across Transitional Mono to M0. (**E**) Kaplan–Meier survival curves comparing overall survival between high and low angiogenesis-related M0 macrophage signature groups in LIHC.

Given that *HBEGF*, *VEGFA*, and members of the CXCL family are well-established key mediators of macrophage-driven angiogenesis, we next investigated the expression pattern of our predefined angiogenesis-related gene set in Transitional Mono to M0 cells via gene set scoring analysis (Figure 3A). We found that *NFKBIZ*^+^ macrophages exhibited a significantly higher angiogenesis score compared with all other Transitional Mono to M0 subsets (Figure 3B). Combined with our previous observation that 84.9% of *NFKBIZ*^+^ macrophages are enriched in the non-pCR group, these data collectively suggest that the angiogenesis gene set expressed by *NFKBIZ*^+^ macrophages may play a key role in mediating ICB resistance in HCC.

To further validate this hypothesis, we performed differential expression analysis of Transitional Mono to M0 cells between the pCR and non-pCR groups. All genes within the angiogenesis-related gene set were consistently and significantly upregulated in the non-pCR group (Figure 3C), with particularly strong differences observed for *VEGFA*, *CXCL8*, *CXCL2*, *CXCL3*, and *HBEGF*. Among them, *VEGFA* and *HBEGF* are known to exert central roles in driving tumor angiogenesis [19,35], while *CXCL8* and its related chemokines positively regulate angiogenic processes [34]. At the individual gene level, apart from these five core angiogenic genes, *FST*, *PDGFB*, and *CXCL1* showed relatively low expression levels in both groups, whereas TGFB1 was highly expressed but exhibited minimal differential expression between pCR and non-pCR groups (Figure 3C,D).

To further validate the clinical relevance of these pro-angiogenic M0 macrophages, we established a marker signature characterizing pro-angiogenic macrophages by combining the M0 macrophage canonical marker *CD68* with the nine genes from our angiogenesis-related gene set. Using data from The Cancer Genome Atlas (TCGA) liver hepatocellular carcinoma (LIHC) cohort, we assessed the correlation between the infiltration level of these pro-angiogenic macrophages and patient overall survival. We observed a significant negative correlation between the infiltration levels of pro-angiogenic macrophages and patient overall survival (Figure 3E), clinically supporting their pro-tumorigenic role in HCC progression.

### 3.4. NFKBIZ^+^ Macrophages May Confer ICB Therapy Resistance by Promoting Tumor Angiogenesis

**Figure 4:**
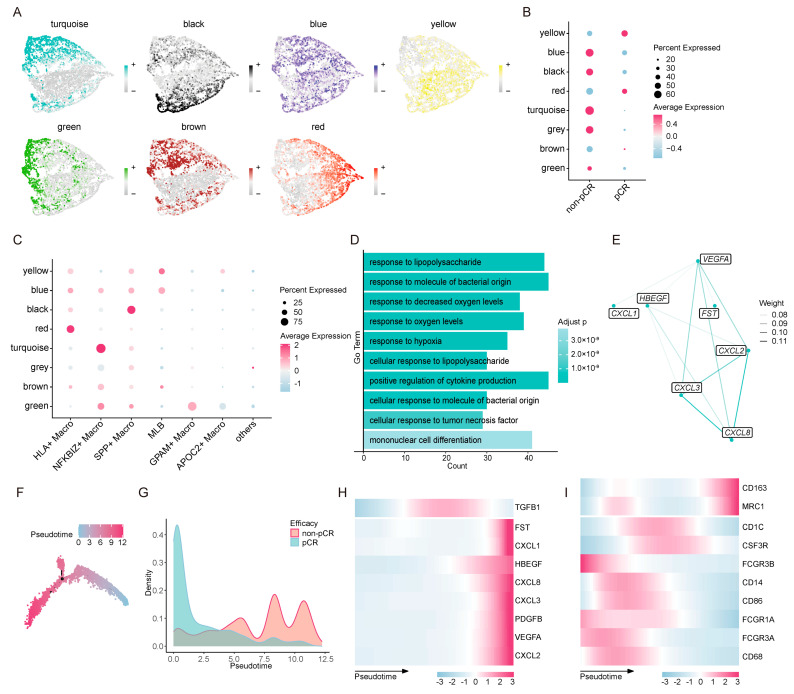
Pseudotime and hdWGCNA analyses reveal angiogenic features of NFKBIZ^+^ macrophages. (**A**) Feature plots showing the expression distribution of each module across Transitional Mono to M0; the gray module (noise) is omitted. (**B**) A dot plot showing the average expression and the percentage of cells expressing each module in the pCR and non-pCR groups. (**C**) A dot plot showing the average expression and the percentage of cells expressing each module across Transitional Mono to M0 subsets. (**D**) A GO-BP enrichment analysis of the turquoise module. (**E**) A weighted gene co-expression network showing the interactions within the angiogenesis-related gene set. (**F**) A diffusion pseudotime plot showing inferred developmental trajectories with pseudotime progression indicated by color gradient. (**G**) Density plots comparing the distribution of pCR and non-pCR cells along pseudotime. (**H**) A heatmap depicting the expression dynamics of angiogenesis-related genes (*VEGFA*, *HBEGF*, *FST*, *PDGFB*, *TGFB1*, *CXCL1*, *CXCL2*, *CXCL3*, and *CXCL8*) along the pseudotime trajectory. (**I**) A heatmap depicting the expression dynamics of monocyte–macrophage differentiation-associated genes (*CD14*, *CD1C*, *FCGR3A*, *FCGR3B*, *CSF3R*, *CD68*, *CD86*, *FCGR1A*, *CD163* and *MRC1*) along the pseudotime trajectory.

To elucidate the co-expression patterns among genes in the angiogenesis-related gene set, we performed high-dimensional weighted gene co-expression network analysis (hdWGCNA) on Transitional Mono to M0 cells. We selected the soft power threshold as recommended by hdWGCNA’s default criteria (Figure S3A), which yielded seven major co-expression modules (Figure 4A and Figure S3B); modules were labeled with distinct colors for purely illustrative purposes. Notably, the results indicated that all nine genes were clustered within the turquoise module, characterized by markedly higher expression and a larger proportion of cells in the non-pCR group. In contrast, the turquoise module showed minimal expression in the pCR group (Figure 4B), highlighting its potential involvement in non-response. Further, the turquoise module was specifically enriched in *NFKBIZ*^+^ macrophages (Figure 4C), which reinforces the pivotal role of this subset in mediating ICB resistance.

Functionally, the turquoise module was primarily involved in biological processes such as inflammatory response, immune regulation, and hypoxia response (Figure 4D). These processes are tightly interconnected in the inflammation–hypoxia–angiogenesis regulatory network, further validating the pro-angiogenic potential of *NFKBIZ*^+^ macrophages. Within the turquoise module, network analysis revealed robust co-expression among the core angiogenic genes *VEGFA*, *CXCL8*, *CXCL2*, *CXCL3*, and *HBEGF* (Figure 4E). Specifically, *CXCL2*, *CXCL3*, and *CXCL8* showed particularly strong correlations with each other and with VEGFA (Figure 4E), highlighting these four genes as the central hub of the angiogenesis-related gene set.

To reveal the gene expression dynamics underlying ICB response, we performed a pseudotime trajectory analysis of Transitional Mono to M0 cells (Figure 4F). The results showed that cells from the pCR group were primarily concentrated in the early stage of the trajectory, while non-pCR cells were predominantly enriched in the middle and late stages (Figure 4G), suggesting that late-stage trajectory states correlate with ICB resistance. Consistent with this, *NFKBIZ*^+^ macrophages exhibited distinct accumulation in the later stages of the pseudotime trajectory, which closely aligned with ICB non-response (Figure S4). Meanwhile, all genes in the angiogenesis-related gene set except *TGFB1* showed high expression in the late trajectory stages (Figure 4H). Notably, the M2 macrophage-associated markers *CD163* and *MRC1* were also upregulated in the late trajectory stages (Figure 4I), suggesting that *NFKBIZ*^+^ macrophages may exhibit a tendency to differentiate into an M2-like macrophage state. Collectively, these data support the notion that *NFKBIZ*^+^ macrophages are associated with an angiogenic and immunosuppressive functional program during the late-stage transitional monocyte-to-M0 state, which may contribute to ICB resistance.

We leveraged IHC datasets from the Human Protein Atlas (HPA) database to further assess the expression of angiogenesis-related protein biomarkers in normal liver tissue, HCC, and cholangiocarcinoma. Notably, *VEGFA* (Figure S5A) and *CXCL8* (Figure S5B) exhibited higher protein expression in HCC and cholangiocarcinoma tumor tissues compared with normal liver tissue. These observations demonstrate an elevated protein expression of *VEGFA* and *CXCL8* in tumor tissues, offering independent protein-level validation for their potential involvement in tumor-associated angiogenic processes.

### 3.5. FOSB–VEGFA and FOS–HBEGF Regulatory Axes Potentially Regulate Angiogenic Activity in NFKBIZ^+^ Macrophages

**Figure 5:**
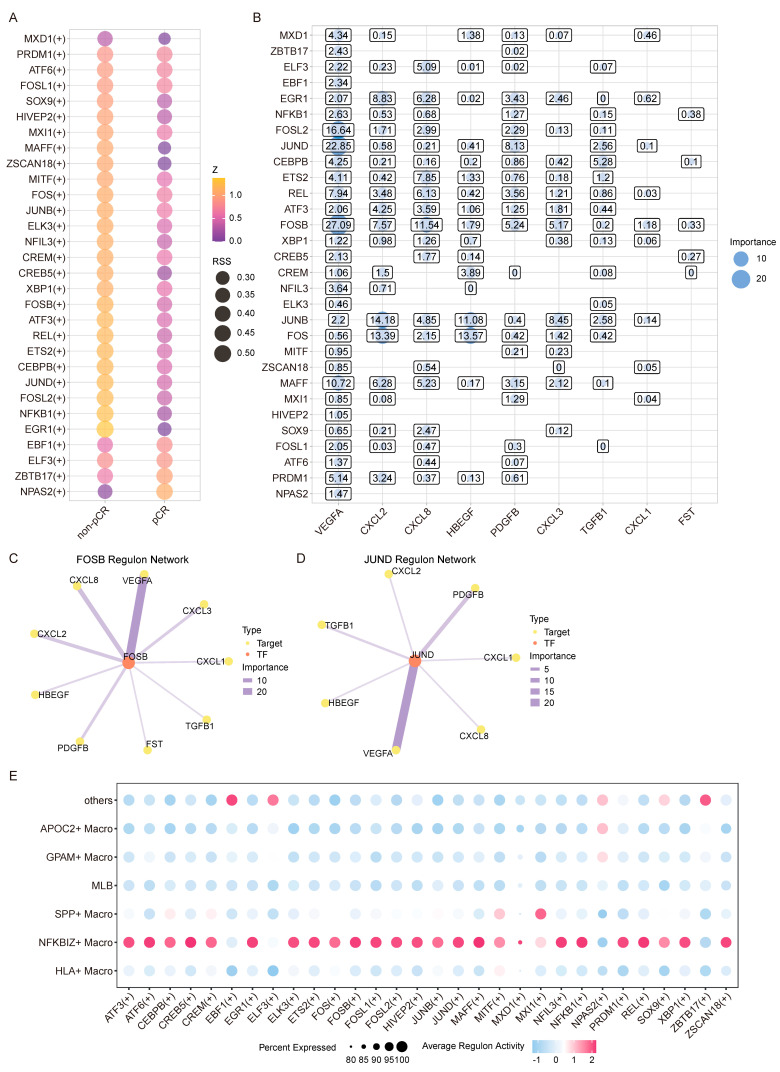
pySCENIC analysis identifies transcription factor networks regulating angiogenesis in NFKBIZ^+^ macrophages. (**A**) A comparison of transcription factor activity scores between the pCR and non-pCR groups using pySCENIC, with dot size representing the regulon specificity score (RSS) and color indicating the Z score. (**B**) A correlation analysis between transcription factor expression and angiogenesis-related gene levels. Dot size represents the correlation coefficient between each TF and target gene. (**C**,**D**) Regulon networks of (**C**) *FOSB* and (**D**) *JUND*. Edge thickness represents the importance score of each TF–target interaction in the GRN inferred by pySCENIC. Node color/size indicates TF or target properties as labeled. (**E**) A dot plot showing regulon AUC scores of transcription factors associated with the angiogenesis-related gene set across Transitional Mono to M0 subsets. Dot size represents the percentage of cells expressing the regulon, and dot color indicates the average regulon activity.

To identify the transcription factor network regulating angiogenesis in *NFKBIZ*^+^ macrophages, we used pySCENIC to predict transcription factor activity from scRNA-seq data of Transitional Mono to M0 cells. This analysis identified 30 potential transcription factors associated with the angiogenesis-related gene set, most of which exhibited stronger activity in the non-pCR group (Figure 5A), which is consistent with the elevated angiogenic signature in ICB non-responders. Notably, *FOSB* and *JUND* showed the strongest correlation with *VEGFA*. Beyond *VEGFA*, *FOSB* also displayed strong associations with *CXCL8* and other key genes in the angiogenesis-related gene set (Figure 5B), highlighting its broad regulatory role in angiogenic signaling. Furthermore, transcription factors *FOS* and *JUNB* showed robust correlations with *CXCL2* and *HBEGF* (Figure 5B), suggesting multiple molecular mechanisms regulating tumor angiogenesis in Transitional Mono to M0 cells.

Consistent with our earlier identification of core angiogenic genes, within FOSB’s regulatory network, *VEGFA*, *CXCL8*, *CXCL2*, and *CXCL3* exhibited stronger associations than other genes in the set (Figure 5C). On the other hand, *JUND* showed prominent correlations with *PDGFB* and *TGFB1* (Figure 5D), indicating the functional specialization of transcription factors within the network. We further analyzed the expression distribution of the transcription factors and found that most were enriched in *NFKBIZ*^+^ macrophages, with higher expression levels and cellular proportions in this subset (Figure 5E). *FOSB* and *JUND* displayed highly consistent expression patterns, overlapping significantly with *NFKBIZ*^+^ macrophages, whereas *FOS* and *JUNB* showed more widespread expression across multiple myeloid subsets (Figure S6). In contrast, transcription factors with low expression in *NFKBIZ*^+^ macrophages (e.g., *NPAS2* and *EBF1*) showed weak associations with the angiogenesis-related gene set (Figure 5B,E), reinforcing a transcription factor expression-dependent regulatory effect.

Collectively, these results indicate that the *FOSB*–*VEGFA* and *FOS*–*HBEGF* axes are enriched in angiogenesis-associated transcriptional programs in *NFKBIZ*^+^ macrophages, highlighting a potential link between this macrophage state and ICB non-responsiveness in HCC.

## 4. Discussion

HCC is the sixth most common malignancy and the third leading cause of cancer-related death worldwide [1]. It accounts for over 80% of primary liver cancers [52] and is strongly associated with chronic liver diseases such as viral hepatitis, alcoholic liver disease, and metabolic-associated fatty liver disease [53]. Despite therapeutic advances, prognosis remains poor due to late diagnosis, high recurrence, and treatment resistance [53]. ICB, especially PD-1/PD-L1 inhibitors, has improved outcomes in some HCC patients by restoring T-cell-mediated antitumor immunity [10,11]. However, only a minority achieve durable responses, as most develop resistance [14,15]. TAMs critically influence ICB efficacy: M1-like TAMs promote antitumor immunity, whereas M2-like TAMs suppress immune activity and impair response [54,55]. Additionally, the infiltration of M0 macrophages correlates with poor prognosis in HCC [26,27].

To explore the impact of M0 macrophage infiltration on the efficacy of immunotherapy in HCC, we analyzed the heterogeneity of myeloid cells in ICB-treated tumors at the single-cell level. Our core finding is the identification of a distinct NFKBIZ^+^ macrophage subset that is significantly enriched in non-responders (non-pCR) and exhibits the highest angiogenesis-related gene scores, suggesting a key role in ICB resistance. Angiogenesis is a hallmark of cancer progression: it not only supplies oxygen and nutrients to tumor cells but also shapes an immunosuppressive microenvironment that promotes immune evasion, driving tumor growth, metastasis, and therapy resistance [40]. Consistent with this, we found all angiogenesis-related genes to be significantly upregulated in non-pCR patients, with *VEGFA*, *CXCL8*, *CXCL2*, *CXCL3*, and *HBEGF* identified as core drivers of tumor angiogenesis and immune escape.

These core genes are likely to act in a coordinated pro-angiogenic manner: *VEGFA* activates *VEGFR2*-mediated PI3K–AKT and ERK signaling to stimulate endothelial cell proliferation, migration, and vascular permeability [19,56,57], supplying critical nutrients and oxygen to support tumor growth; CXCL family chemokines (*CXCL8*, *CXCL2*, and *CXCL3*) indirectly promote angiogenesis by enhancing inflammatory responses [58] and activating endothelial cell functions [59]; and *HBEGF*, an important ligand of the epidermal growth factor receptor (EGFR) family, potently stimulates endothelial cell proliferation, tube formation, and vascular permeability, even at low concentrations [35]. Their coordinated upregulation in *NFKBIZ*^+^ macrophages highlight a convergent mechanism underlying pathological angiogenesis in resistant tumors.

High-dimensional weighted gene co-expression network analysis (hdWGCNA) further revealed that these core angiogenic genes cluster within a “turquoise” module specifically expressed in *NFKBIZ*^+^ macrophages and strongly associated with ICB non-response. This module is enriched in pathways related to inflammation, hypoxia adaptation, and immune regulation, positioning *NFKBIZ*^+^ macrophages as a key component in an inflammation–hypoxia–angiogenesis regulatory network. This finding provides insight into how *NFKBIZ*^+^ macrophages integrate microenvironmental cues (e.g., hypoxia) to amplify pro-angiogenic and immunosuppressive signaling, creating a feedforward loop that promotes immune escape and tumor progression.

Transcription factor analysis revealed the candidate molecular regulators of this angiogenic program: *FOSB* emerged as the most prominent regulator of the angiogenesis-related gene set, with strong correlations not only with *VEGFA* but also with *CXCL8*, *CXCL2*, and *CXCL3*, underscoring its broad control over angiogenic and inflammatory signaling. In addition, *FOS* exhibited a robust connection with *HBEGF*, suggesting a cooperative regulatory mechanism wherein *FOSB*–*VEGFA* and *FOS*–*HBEGF* axes synergistically upregulate angiogenic gene expression in *NFKBIZ*^+^ macrophages. This co-regulation of *VEGFA* and *HBEGF* likely enhances vascular remodeling and tumor progression through complementary pathways, further reinforcing ICB resistance.

Clinically, our findings have important implications for overcoming angiogenesis-driven ICB resistance. Currently, anti-angiogenic therapies targeting *VEGFA* (e.g., bevacizumab) in combination with PD-L1 inhibitors are first-line therapy for advanced HCC [60]. However, *VEGFA* inhibition has been reported to induce the compensatory upregulation of *HBEGF*, limiting therapeutic efficacy. Conversely, *HBEGF* depletion can simultaneously downregulate *VEGFA* and markedly suppress tumor growth [61], indicating that *HBEGF* may be a more effective therapeutic target to disrupt this pro-angiogenic network. Notably, no HCC treatments specifically targeting *HBEGF* are currently available, making it a promising and more effective therapeutic target for overcoming angiogenesis-driven resistance and improving immunotherapy outcomes.

## 5. Conclusions

This study identifies a previously unrecognized pro-angiogenic macrophage subset—*NFKBIZ*^+^ macrophages—that critically contributes to ICB resistance in HCC. We demonstrate that these macrophages are preferentially enriched in non-responders, characterized by the coordinated upregulation of *VEGFA*, CXCL family chemokines, and *HBEGF*, and driven by the *FOSB*–*VEGFA* and *FOS*–*HBEGF* transcriptional axes.

These findings clarify how myeloid cell heterogeneity shapes ICB outcomes in HCC and highlight the *VEGFA*–*HBEGF* axis as a central conduit for angiogenesis-driven immunosuppression. Targeting *HBEGF*—alone or in rational combination with ICB—may offer a more effective strategy to disrupt this network and enhance the durability of immunotherapy responses.

Collectively, our work advances our understanding of macrophage-mediated ICB resistance and provides a conceptual and molecular framework for developing next-generation therapeutic strategies to overcome angiogenesis-associated immune escape in HCC.

## Data Availability

Data included in this paper were acquired from public repositories, including the European Genome-phenome Archive under accession number EGAS00001007547 and the National Center for Biotechnology Information’s Gene Expression Omnibus (GSE206325). TCGA gene expression and clinical data used in this study can be accessed via the Xena Browser (https://xenabrowser.net/datapages/), accessed on 2 December 2024. No algorithm or software was generated for this study. The workflow code employed in this study is available in the Zenodo repository at the following DOI: 10.5281/zenodo.17549380. The repository contains the scripts necessary to reproduce the analyses conducted in this research.

## Supplementary Materials

The following supporting information can be downloaded at: https://www.mdpi.com/article/10.3390/biology15010095/s1, Figure S1: Feature plots showing the expression distribution of representative marker genes (*CD14*, *CD1C*, *FCGR3A*, *FCGR3B*, *CSF3R*, *CD68*, *CD86*, *FCGR1A*, *CD163*, and *MRC1*) across myeloid cells; Figure S2: Optimization and quality assessment of NMF components; Figure S3: hdWGCNA analysis of Transitional Mono to M0 cells; Figure S4: Pseudotime trajectories colored by cell types: *HLA*^+^ Macro, *NFKBIZ*^+^ Macro, *SPP*^+^ Macro, MLB, *GPAM*^+^ Macro, and *APOC2*^+^ Macro; Figure S5: Immunohistochemical validation of *VEGFA* and *CXCL8* expression in liver cancers; Figure S6: Distribution of regulon AUC scores for 30 potential transcription factors associated with the angiogenesis-related gene set across Transitional Mono to M0.

## Abbreviations

HCC, hepatocellular carcinoma; ICB, immune checkpoint blockade; TME, tumor microenvironment; PD-1, programmed cell death protein 1; PD-L1/PD-L2, programmed death-ligand 1/2; TAMs, tumor-associated macrophages; M0/M1/M2, macrophage polarization states (unpolarized/classically activated/alternatively activated); *NFKBIZ*, nuclear factor κB inhibitor zeta; *VEGFA*, vascular endothelial growth factor A; *HBEGF*, heparin-binding EGF-like growth factor; CXCL, C-X-C motif chemokine ligand; *FOSB*, FosB proto-oncogene, AP-1 transcription factor subunit; *FOS*, Fos proto-oncogene, AP-1 transcription factor subunit; *JUND*, JunD proto-oncogene, AP-1 transcription factor subunit; *JUNB*, JunB proto-oncogene, AP-1 transcription factor subunit; scRNA-seq, single-cell RNA sequencing; cNMF, consensus non-negative matrix factorization; hdWGCNA, high-dimensional weighted gene co-expression network analysis; GRN, gene regulatory network; GO, Gene Ontology; BP, biological process; TF, transcription factor; DEG, differentially expressed gene; FDR, false discovery rate; ssGSEA, single-sample gene set enrichment analysis; GSVA, gene set variation analysis; pCR, pathological complete response; *VEGFR2*, vascular endothelial growth factor receptor 2; EGFR, epidermal growth factor receptor; LIHC, liver hepatocellular carcinoma (TCGA dataset); TOM, topological overlap matrix; UMAP, uniform manifold approximation and projection.
