# Supplementary material for: Single-Cell Transcriptomic Profiling Reveals That Macrophage-Induced Angiogenesis Contributes to Immunotherapy Resistance in Hepatocellular Carcinoma
Source: Biology (Basel). 2026 Jan 2;15(1):95. doi: 10.3390/biology15010095 (PMC12784835; doi:10.3390/biology15010095)
Supplement: Supplementary file 1 [file biology-15-00095-s001.zip › Supplementary Materials.pdf]

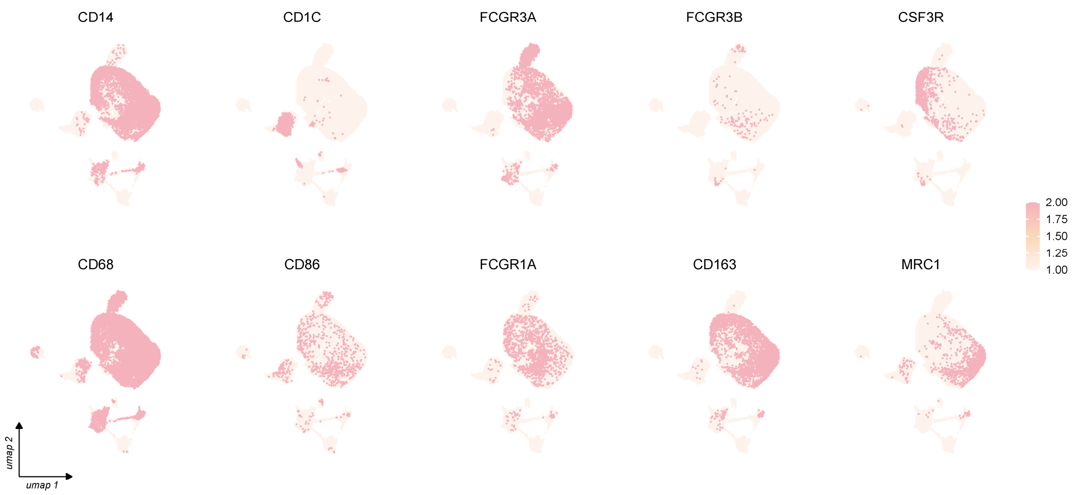

Figure S1. Feature plots showing the expression distribution of representative marker genes (CD14, CD1C, FCGR3A, FCGR3B, CSF3R, CD68, CD86, FCGR1A, CD163, and MRC1) across myeloid cells.

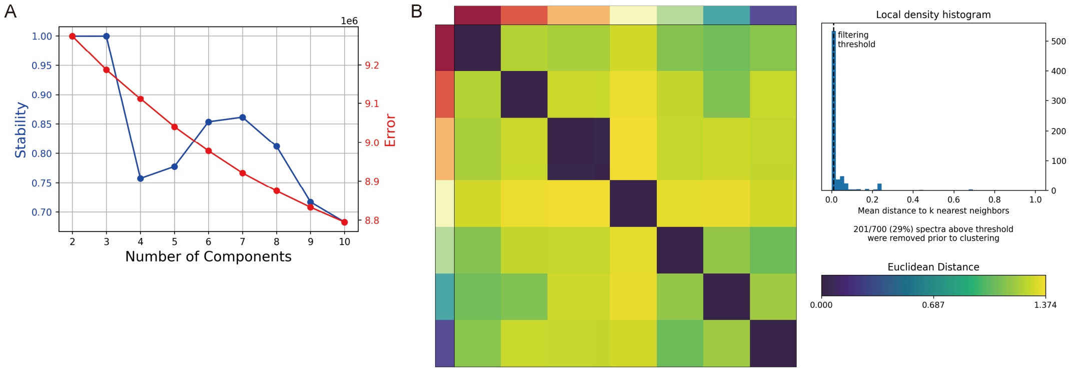

Figure S2. Optimization and quality assessment of NMF components. A: Plot of stability (blue) and error (red) used to determine the optimal number of NMF components. B: Left: Heatmap of component similarity, demonstrating clustering quality. Right: Local density histogram showing thresholding criteria for cluster separation based on Euclidean distance.

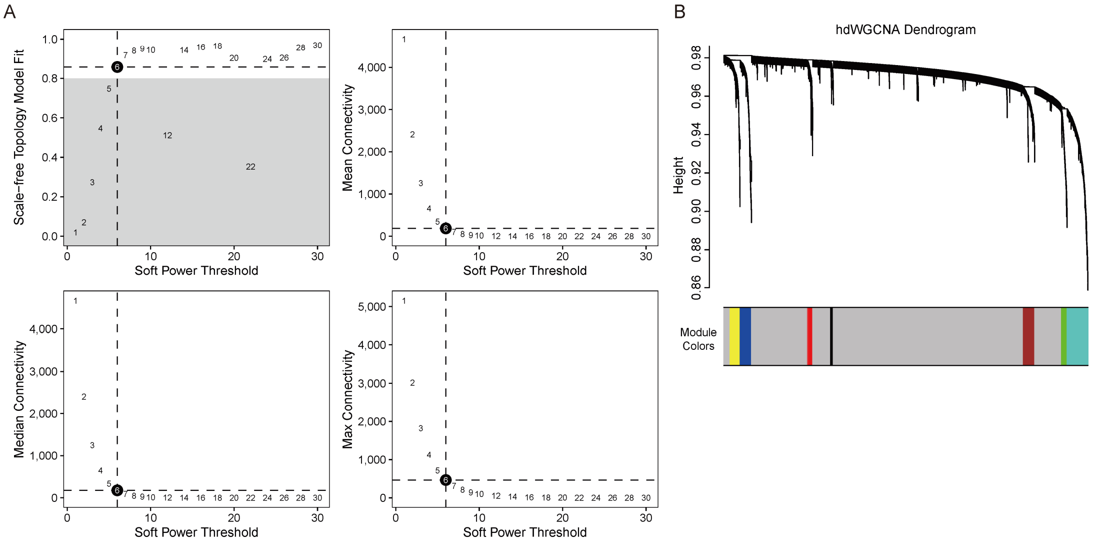

Figure S3. hdWGCNA analysis of Transitional Mono to M0 cells. A. Selection of the soft-thresholding power for hdWGCNA. Plots show scale-free topology model fit, mean, median, and maximum connectivity across a range of soft power thresholds; the chosen threshold (6) is indicated. B. Dendrogram of gene modules identified by hdWGCNA. Modules are color-coded.

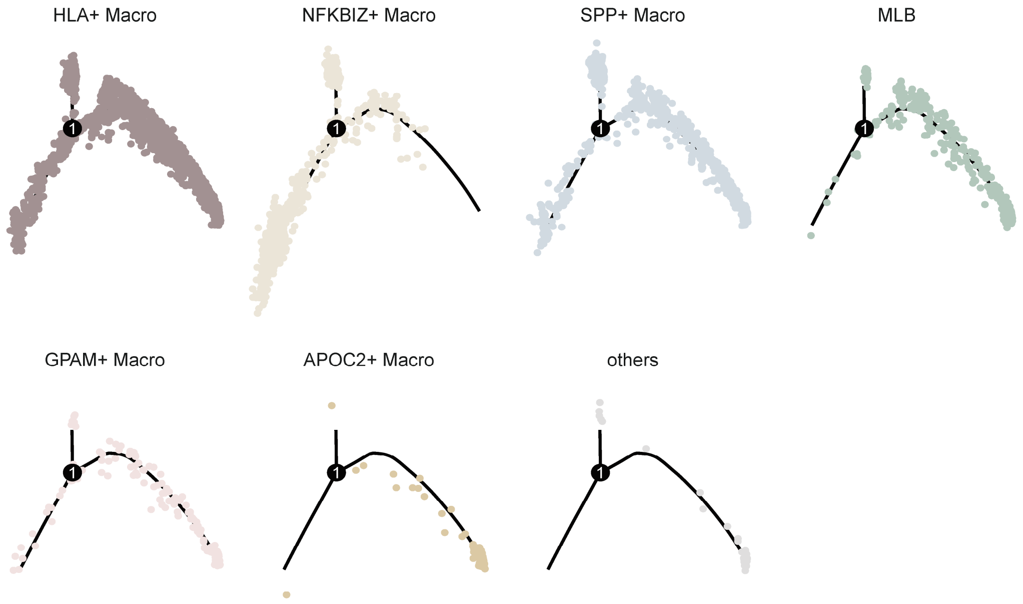

Figure S4. Pseudotime trajectories colored by cell types: HLA⁺ Macro, NFKBIZ⁺ Macro, SPP⁺ Macro, MLB, GPAM⁺ Macro, and APOC2⁺ Macro.

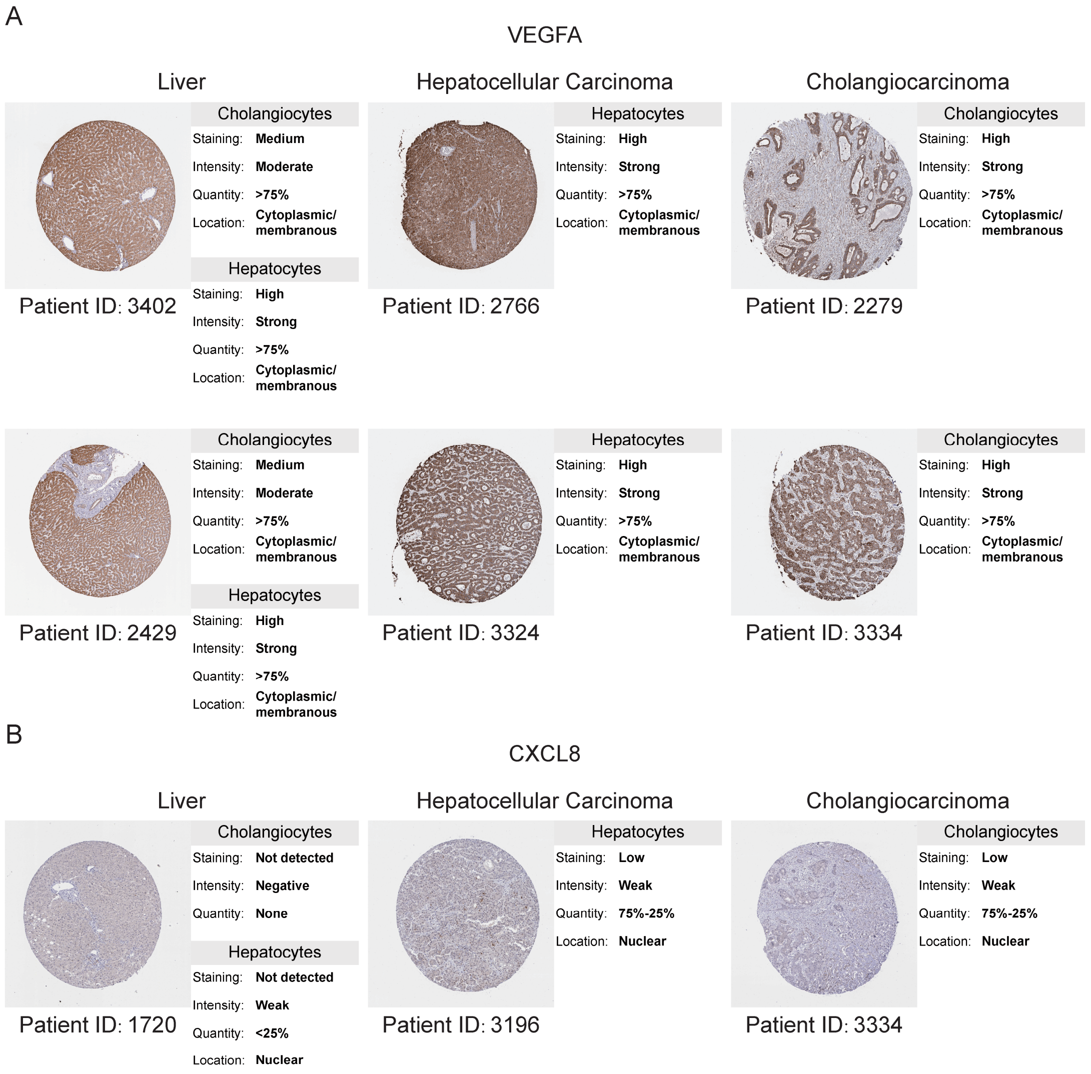

Figure S5. Immunohistochemical validation of VEGFA and CXCL8 expression in liver cancers. Representative IHC images obtained from the HPA database showing VEGFA (A) and CXCL8 (B) protein expression in normal liver tissue, HCC, and cholangiocarcinoma.

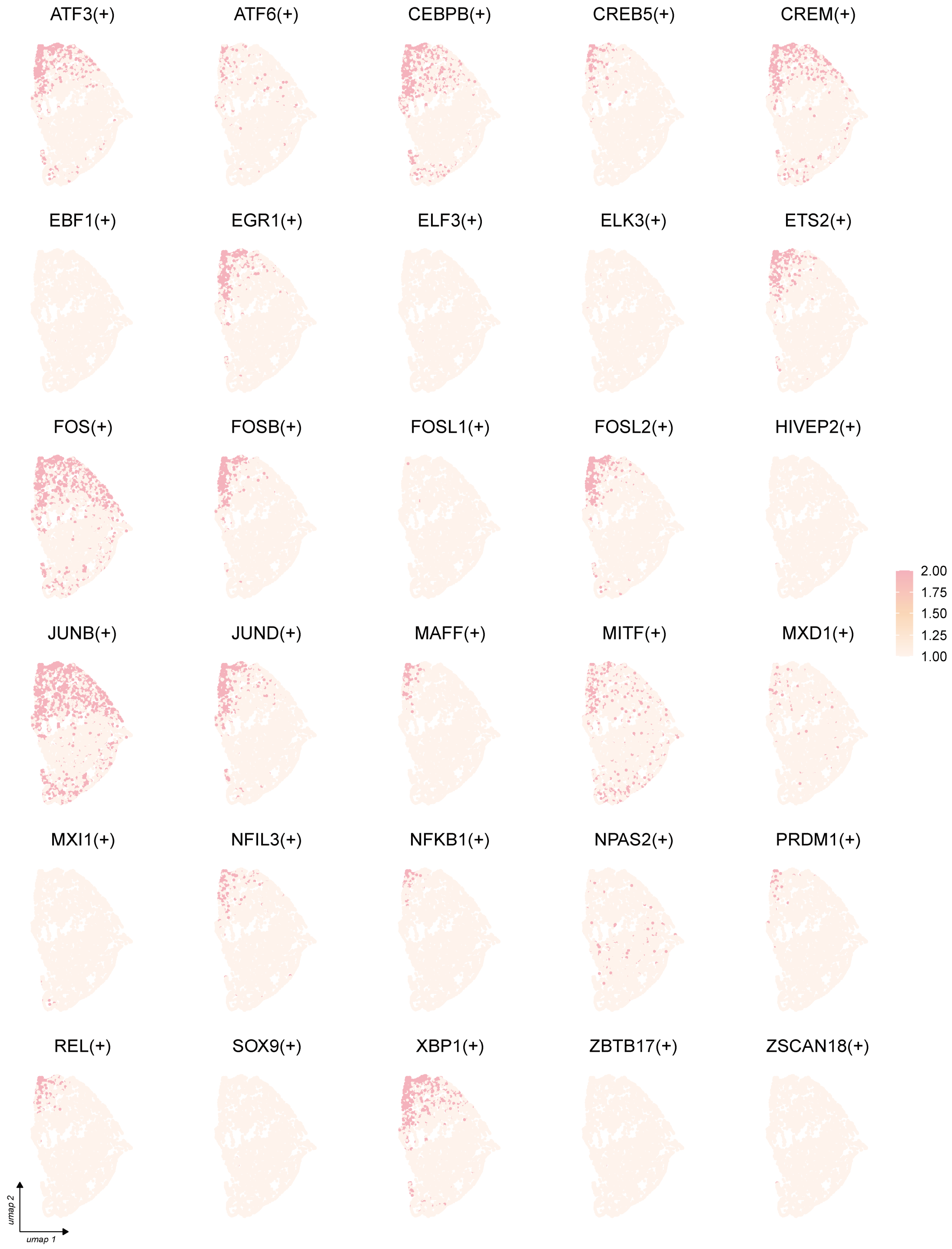

Figure S6. Distribution of regulon AUC scores for 30 potential transcription factors associated with the angiogenesis-related gene set across Transitional Mono to M0.
